# 

**Published:** 1840

**Authors:** 


### CONTENTS OF NOS. VII & VIII.

ART. I. An Investigation on the Nature, Growth, and Formation of the Human Teeth, with an explanation of the causes of their decay; particularly of its uncommon prevalence in the middle and northern States of America.—By Horace H. Hayden, M. D. of Baltimore—page 137.

ART. II. Minutes of the Convention Assembled in the city of New-York, on the 18th day of August, 1840, for the purpose of forming a National Dentists' Society, together with an account of the organization of said Society, with its Constitution and By-Laws—page 157.

ART. III. Treatment of the Nerves of the Teeth. By E. Baker—page 170.

ART. IV. Galvanic Action of Metals in the Mouth. By J. Brockway—172.

ART. V. Case of Irregular arrangement, and the remedy. By B. A. Rodriguez, M. D.—page 175.

ART. VI. Case of Exostosis, and Osseous Union of two molar Teeth. By G. Merryman,—page 176.

ART. VII. Notice of the American Society of Dental Surgeons,—page 177.

ART. VIII. Bibliography.—page 180.

ART. IX. Notice to Correspondents,—page 184.

ART. X. Miscellaneous,—page 184.

